# Transient Supplement-Associated Thyrotoxicosis Without Persistent Hyperthyroidism: A Diagnostic Challenge in Endocrine Practice

**DOI:** 10.14740/jmc5345

**Published:** 2026-06-03

**Authors:** Pavel Stanchev, Ekaterina Babadzhanova, Boris Tilov, Maria Kraeva, Delyana Davcheva

**Affiliations:** a Department and Clinic of Endocrinology and Metabolic Diseases, St. George University Hospital, Medical University of Plovdiv, Plovdiv, Bulgaria; b Medical College, Medical University of Plovdiv, Plovdiv, Bulgaria; c Department of Otorhinolaryngology, Medical Faculty, Medical University of Plovdiv, Plovdiv, Bulgaria; d Department of Clinical Laboratory, Medical Faculty, Medical University of Plovdiv, Plovdiv, Bulgaria

**Keywords:** Thyrotoxicosis, Hyperthyroidism, Iodine

## Abstract

Thyrotoxicosis refers to the clinical state resulting from elevated thyroid hormone levels and may occur with or without underlying hyperthyroidism. Recognizing non-thyroidal and iatrogenic causes is essential for accurate diagnosis and management. A 50-year-old woman presented to a cardiologist due to complaints of palpitations. Electrocardiogram and echocardiogram were performed showing sinus tachycardia. Beta-blocker therapy was prescribed with recommendations for thyroid function testing. The laboratory tests performed in a specialized endocrine clinic confirmed transient thyrotoxicosis without hyperthyroidism and a normal ultrasound image of the thyroid gland. Upon further assessment, an iatrogenic cause was suspected as the most plausible contributing factor—the use of a nutritional supplement for menopausal symptoms containing iodine and other active substances. We present a clinical case of transient most likely supplement-associated thyrotoxicosis without persistent hyperthyroidism with subsequent normalization of thyroid function after symptomatic therapy. Exogenous iodine intake in the form of a dietary supplement is discussed as a possible trigger. The article provides information about the diagnostic and therapeutic algorithm in patients with thyrotoxicosis in the light of real clinical practice and emphasizes the importance of detailed medication and supplement history.

## Introduction

Thyrotoxicosis is a syndrome caused by an excess of free thyroid hormones. Symptoms depend on the degree of hormone elevation, the duration of exposure, the rate at which hormone levels have increased, and the individual characteristics of the patients [1].

The term thyrotoxicosis is not synonymous with hyperthyroidism—elevation in thyroid hormone levels caused by an increase in their biosynthesis and secretion [2]. For example, thyrotoxicosis may result from the destruction of thyroid follicles and thyrocytes in various forms of thyroiditis or may be caused by excessive intake of exogenous thyroid hormones—thyrotoxicosis without hyperthyroidism. It should also be noted that an increase in free thyroid hormone levels does not always lead to thyrotoxicosis in all tissues.

Usually, cases of thyrotoxicosis without hyperthyroidism are transient in nature. Etiologically, the following causes of thyrotoxicosis without hyperthyroidism are encountered: destructive thyroiditis, subacute thyroiditis, painless thyroiditis, ectopic thyroid tissue, and thyrotoxicosis due to exogenous substances(iodine-based and thyrotoxicosis factitia) [3]. Iodine-induced thyrotoxicosis can result from excess iodine intake, which leads to uncontrolled release of preformed thyroid hormones. Sources of excess iodine that can lead to this condition include dietary supplements containing iodine (e.g. seaweed-based products), medications, particularly amiodarone and iodine-containing multivitamins, iodinated contrast agents used in imaging procedures like computed tomography (CT) scans or angiography, and topical antiseptics containing povidone-iodine, which highlights the need to always keep iodine in mind as a potential cause of thyrotoxicosis.

We present a clinical case of transient thyrotoxicosis without persistent hyperthyroidism in a 50-year-old woman with a normal ultrasound image and recovery of thyroid function without specific antithyroid treatment.

## Case Report

### Investigations

A 50-year-old woman consulted a cardiologist regarding complaints of palpitations. She reported no chronic diseases or medication intake. She shared with the medical team for the presence of amenorrhea for about 6 months, and given her age and likely the onset of menopause, a consultation with a gynecologist was conducted, with no evidence of pathological findings regarding the uterus and the ovaries. The patient had evidence of sinus tachycardia with no data on pathological conditions from the performed electrocardiogram and echocardiography. Beta-blocker therapy was prescribed and a recommendation for thyroid function testing was given. The conducted hormonal tests showed evidence of thyrotoxicosis,– and the patient was referred for diagnostic clarification to a specialized endocrine clinic.

### Diagnosis

Physical examination showed warm and moist skin with no abnormalities of the head and neck. The patient presented with a normosthenic habitus, with height of 170 cm, weight of 64 kg, and body mass index (BMI) of 22.1 kg/m^2^. Thyroid gland was not palpable and not enlarged. No pathology in the peripheral lymph nodes was observed. Vesicular breathing bilaterally, without wheezing. Rhythmic cardiac activity with a frequency of 82 beats/min and arterial pressure of 110/60 mm Hg. No heart murmurs were detected by auscultation. Abdomen was soft, painless with physiological peristalsis. Liver and spleen were not palpable. There was no evidence of edema on the lower limbs or varicose changes, and the peripheral pulsations on the lower limbs were palpable.

Following a detailed medical history, the patient reported regular intake of a dietary supplement for a few months intended for menopausal symptom relief, which she discontinued after the onset of palpitations. The reported regimen was 3 tablets per day. Each tablet contained 10.5 µg of iodine, totaling 31.5 µg daily. Although the other listed ingredients were not primarily associated with thyroid function, the potential contribution of combined components to the observed symptoms cannot be entirely excluded. Some of them included biotin (20 µg per capsule), which, while not directly causing thyrotoxicosis, may interfere with thyroid immunoassays, licorice root, Siberian ginseng, chasteberry, *Angelica sinensis*, blessed thistle, and black cohosh, all of which may exhibit phytoestrogenic or immunomodulatory properties.

From the studies conducted, the patient presented with data on thyrotoxicosis with negative thyroid autoantibodies, normal blood count, inflammatory markers, ionogram, and renal function (Table 1).

**Table 1:** Basic Investigations of Thyroid Function Tests Demonstrating Thyrotoxicosis (Suppressed TSH, Elevated FT4 and FT3)

Lab test	Value	Reference range
Hemoglobin	132	120–160 g/L
CRP	4.1	0–10 mg/L
Fasting glucose	5.31	2.8–6.1 mmol/L
Serum potassium	4.6	3.5–5.6 mmol/L
Serum sodium	139.7	136–151 mmol/L
Serum chlorine	101.2	96–110 mmol/L
Urea	4.8	2.6–7.2 mmol/L
Creatinine	78.5	44–96 µmol/L
TSH	0.005↓	0.34–5.6 mU/L
FT4	36.85↑	7.86–14.41 pmol/L
FT3	14.13↑	3.8–6.0 pmol/L
Anti-TgAb	2.53	0–4 IU/mL
Anti-TPO-Ab	0.26	0–9 IU/mL
TRAb	0.806	0–1.5 IU/L
Tg	67.4	3.5–77 ng/mL

Anti-TgAb: anti-thyroglobulin antibody; Anti-TPO-Ab: anti-thyroperoxidase antibody; CRP: C-reactive protein; FT3: free triiodothyronine; FT4: free thyroxine; Tg: thyroglobulin; TRAb: TSH receptor antibody; TSH: thyroid-stimulating hormone.

**Figure 1:**
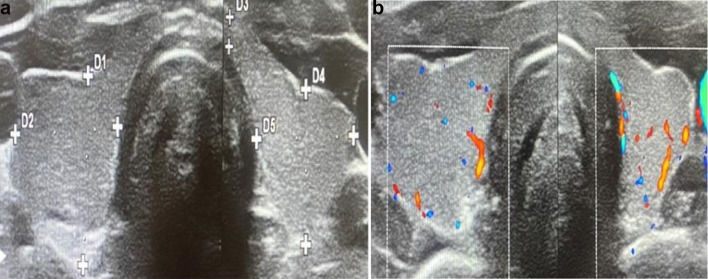
(a) Ultrasound examination of the thyroid gland with data on normal shape, size, localization, and ultrasound structure of the parenchyma. (b) Color Doppler with data on normal blood supply.

The conducted ultrasound examination showed normal shape, size, localization, and structure of the parenchyma, without nodular formations. No pathologically transformed lymph nodes in the cervical region were visualized. Color Doppler showed normal blood supply to the thyroid gland (Fig. 1).

**Figure 2:**
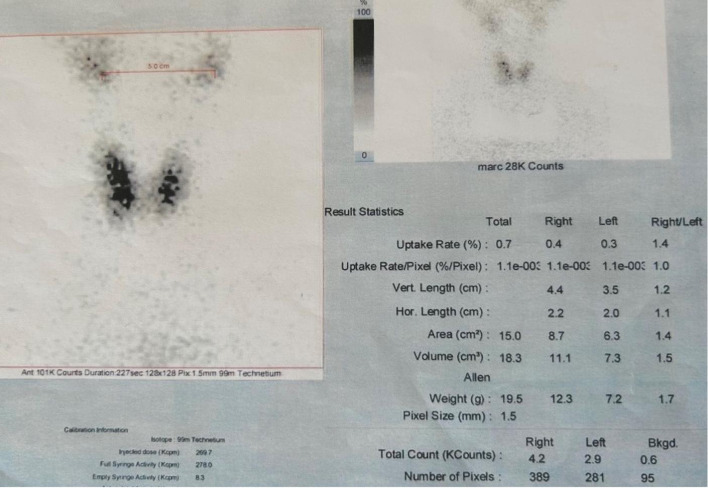
Thyroid scintigraphy using 99m technetium pertechnetate (thyroid scan) approximately 30 days after the initial biochemical diagnosis. Normal uptake of the marker was seen in both lobes of the thyroid gland.

The normal ultrasonographic findings together with negative thyroid-stimulating hormone (TSH)-receptor antibodies, normal Doppler vascularity, and spontaneous recovery made Graves’ disease unlikely. Toxic adenoma and toxic multinodular goiter were also considered unlikely due to the absence of nodular lesions and the transient clinical course. The absence of neck pain and normal inflammatory markers argued against subacute thyroiditis. Painless thyroiditis could not be completely excluded despite the absence of thyroid autoantibodies and normal ultrasonographic findings. There was no history of levothyroxine intake, and thyroglobulin levels were not suppressed, making thyrotoxicosis factitia less likely. Thyrotoxicosis without hyperthyroidism was observed and the patient was referred for thyroid scintigraphy (thyroid scan), which was performed approximately 30 days after the diagnosis of thyrotoxicosis (Fig. 2).

### Follow-up and outcomes

The establishment of a normal scintigraphic image about 30 days after the diagnosis of thyroid dysfunction confirmed the presence of thyrotoxicosis without hyperthyroidism and the transient nature of the disorder. The patient was treated with a beta-blocker and normalization of thyroid function was established over time (Table 2). A short-term phase of subclinical hypothyroidism was registered without the need of replacement treatment. Subsequent normalization of the thyroid function was observed.

**Table 2:** Hormone Results Over Follow-Up Showing Transient Subclinical Hypothyroidism Observed and the Following Normalization of Thyroid Function

Lab test	First	After 60 days	After 90 days	Reference range
TSH	0.005 ↓	9.689	4.468	0.34–5.6 mU/L
FT4	36.85 ↑	9.48	11.78	7.86–14.41 pmol/L
FT3	14.13 ↑	3.89	4.58	3.8–6.0 pmol/L

FT3: free triiodothyronine; FT4: free thyroxine; TSH: thyroid-stimulating hormone.

We present a rare clinical case of a woman with transient thyrotoxicosis without hyperthyroidism with normal echographic and scintigraphic examinations with subsequent normalization of thyroid function after symptomatic therapy. Antithyroid treatment was not initiated because there was no evidence of persistent thyroid hormone overproduction, Graves’ disease or toxic nodular disease, and the clinical course suggested a transient process. Symptomatic treatment with beta-blocker therapy and close biochemical monitoring were considered appropriate.

To improve clarity regarding the chronology of symptom onset, supplement exposure, biochemical evaluation, imaging studies, treatment, and recovery, a timeline of the clinical course is presented in Table 3.

**Table 3:** Timeline of Clinical Presentation, Investigations, Management, and Follow-Up

Time from symptom onset	Clinical event
Approximately 2 months before presentation	Initiation of menopausal dietary supplement
Day 0	Onset of palpitations and cardiology evaluation; sinus tachycardia documented
Days 1–3	Thyroid function tests demonstrated thyrotoxicosis (suppressed TSH, elevated FT4 and FT3)
Shortly after diagnosis	Endocrinology consultation and discontinuation of dietary supplement
Initial endocrine evaluation	Normal thyroid ultrasonography and Doppler examination; negative thyroid autoantibodies
Approximately day 30	Thyroid scintigraphy with technetium-99m pertechnetate showed normal uptake
Approximately day 60	Transient subclinical hypothyroidism observed during follow-up
Approximately day 90	Normalization of thyroid function tests and clinical recovery

FT3: free triiodothyronine; FT4: free thyroxine; TSH: thyroid-stimulating hormone.

## Discussion

Through this clinical case, we present in a practical and simplified way the algorithm that should be followed by every clinician in cases of thyrotoxicosis of unclear etiology. Graves’ disease and autoimmune thyroiditis (normal ultrasound image, absence of thyroid autoantibodies) were ruled out. Subacute thyroiditis (absence of pain syndrome and elevated inflammatory markers) and silent thyroiditis (absence of ultrasound changes and thyroid autoantibodies) were excluded. There was no history of on the intake of levothyroxine preparation, therefore thyrotoxicosis factitia was ruled out. As an etiological cause, the intake of a dietary supplement, containing iodine and numerous herbal and micronutrient components, with a subsequent manifestation of transient thyrotoxicosis without hyperthyroidism, was discussed with high probability.

Iodine is a mineral element found in minimal amounts in the human body (15–20 mg) and the main biological role of iodine is its participation in the synthesis of thyroid hormones (triiodothyronine (T3) and thyroxine (T4)). The recommended daily intake of iodide to achieve physiological hormone synthesis is 150 µg for adolescents and adults [4].

Depending on the amount of iodine ingested, different changes in thyroid function are observed; low doses of iodine are usually not relevant to thyroid hormone synthesis, while medium and high doses of iodine can cause functional disorders, both in the direction of hypothyroidism and thyrotoxicosis [5].

Several drugs and food preservatives contain large amounts of iodine, which is absorbed directly or indirectly after drug metabolism. Various multivitamin preparations contain iodine in an average concentration of 150 µg, a dose that is considered to reflect the recommended daily intake of iodine. Iodine is also found in large amounts in radiocontrast agents. There is a risk of thyroid dysfunction, including hyperthyroidism and hypothyroidism, after the administration of iodinated contrast agents [6].

Although the temporal relationship between supplement intake and the onset of thyrotoxicosis suggests a possible association, the reported iodine exposure (31.5 µg/day) was below the recommended daily iodine intake for adults and substantially lower than doses typically associated with iodine-induced thyrotoxicosis. Therefore, iodine alone may not fully explain the biochemical abnormalities observed in this patient. Synergistic endocrine effects of herbal components, or contamination of the supplement with thyroid-active substances cannot be entirely excluded. Since the composition of the supplement was based solely on manufacturer labeling and was not independently verified, the possibility of unrecognized iodine excess or thyroid hormone contamination remains a limitation of the present report.

Several herbal components contained in the supplement, including black cohosh, licorice root, Siberian ginseng, chasteberry, and *Angelica sinensis*, have been reported to possess phytoestrogenic, immunomodulatory, or endocrine-modulating properties. Although direct evidence linking these substances to thyrotoxicosis is limited, potential synergistic effects on thyroid physiology or immune regulation cannot be excluded.

Matsubara et al described an outbreak of thyrotoxicosis without hyperthyroidism in the city of Matsuyama, Japan, localized as a small community in the local hospital. One hundred fifty-nine cases of both hospitalized patients and hospital staff were identified. The registered thyrotoxicosis was transient in nature and no antithyroid medications were administered. In 24 patients, thyroid ultrasound was performed with data of normal ultrasound image. Thyroid autoantibodies were also not detected. In 18 patients, thyroid scintigraphy was performed with data of decreased uptake with subsequent normalization after 10 days. No etiological cause for the thyrotoxicosis epidemic had been established; intake of an exogenous substance, a food ingredient used in the local hospital lunch menu, was discussed [6].

The case described by us shares several similarities with the report by Matsubara et al, including transient thyrotoxicosis, absence of thyroid autoantibodies, normal ultrasonographic findings, and spontaneous recovery without antithyroid treatment. However, unlike the outbreak described by Matsubara et al, our patient had a clear temporal relationship between the intake of a menopausal dietary supplement and the onset of symptoms. Although causality cannot be definitively established, the supplement may be considered a plausible contributing factor. Toxic nodular disease appeared unlikely given the normal ultrasonographic and scintigraphic findings and the transient clinical course.

The use of dietary supplements was discussed as a plausible trigger. From the ingredients in the content only iodine with a possible synergistic effect of the other herbal additives may be related to the induction of functional disorders of the thyroid gland.

Recent literature has increasingly emphasized the role of dietary supplements and laboratory assay interference in the differential diagnosis of thyrotoxicosis. The 2016 American Thyroid Association guidelines underline the importance of thyroid autoantibodies, ultrasonography, and scintigraphy in distinguishing hyperthyroidism from transient thyrotoxicosis without persistent hormone overproduction [7]. Piketty et al demonstrated that clinically significant biotin interference with thyroid immunoassays is typically associated with high-dose biotin supplementation, substantially exceeding the amount reported in our patient [8]. Reviews on nutraceutical-associated thyroid dysfunction have also highlighted the growing number of cases related to iodine-containing supplements and herbal preparations with potential endocrine effects [9]. Furthermore, iatrogenic thyrotoxicosis remains an important diagnostic consideration in patients with unexplained transient thyroid dysfunction and exposure to non-prescription preparations [10].

### Conclusion

Knowledge of the main characteristics of transient thyrotoxicosis without hyperthyroidism—transience in functional thyroid tests, high frequency of normal ultrasound image, negative thyroid autoantibodies—would contribute to making correct therapeutic decisions, even in the absence of a clearly established etiological cause. The described clinical case illustrates the diagnostic challenges and highlights the importance of obtaining detailed history, including supplement intake. It provides an important example for the clinical practice, supporting awareness of iatrogenic causes and avoiding unnecessary treatment.

## Data Availability

The data supporting the findings of this study are available from the corresponding author upon reasonable request.
